# Intrinsic resistance to ROS1 inhibition in a patient with *CD74‐ROS1* mediated by AXL overexpression

**DOI:** 10.1111/1759-7714.15116

**Published:** 2023-09-19

**Authors:** Tara L. Peters, Nan Chen, Logan C. Tyler, Anh T. Le, Anastasios Dimou, Robert C. Doebele

**Affiliations:** ^1^ Enliven Therapeutics, Inc. Boulder Colorado USA; ^2^ Division of Medical Oncology University of Colorado Anschutz Medical Campus Aurora Colorado USA; ^3^ OnKure Therapeutics, Inc. Boulder Colorado USA; ^4^ Cell Technologies Shared Resources University of Colorado Anschutz Medical Campus Aurora Colorado USA; ^5^ Division of Medical Oncology Mayo Clinic College of Medicine Rochester Minnesota USA

**Keywords:** AXL, drug resistance, NSCLC, ROS1

## Abstract

**Background:**

The vast majority of patients with ROS1 positive non‐small cell lung cancer (NSCLC) derive clinical benefit from currently approved ROS1 therapies, including crizotinib and entrectinib. However, a small proportion of patients treated with ROS1 inhibitors fail to derive any clinical benefit and demonstrate rapid disease progression. The biological mechanisms underpinning intrinsic resistance remain poorly understood for oncogene‐driven cancers.

**Methods:**

We generated a patient‐derived cell line, CUTO33, from a ROS1 therapy naive patient with CD74‐ROS1+ NSCLC, who ultimately did not respond to a ROS1 inhibitor. We evaluated a panel of ROS1+ patient‐derived NSCLC cell lines and used cell‐based assays to determine the mechanism of intrinsic resistance to ROS1 therapy.

**Results:**

The CUTO33 cell line expressed the *CD74‐ROS1* gene fusion at the RNA and protein level. The ROS1 fusion protein was phosphorylated at baseline consistent with the known intrinsic activity of this oncogene. ROS1 phosphorylation could be inhibited using a wide array of ROS1 inhibitors, however these inhibitors did not block cell proliferation, confirming intrinsic resistance in this model and consistent with the patient's lack of response to a ROS1 inhibitor. CUTO33 expressed high levels of AXL, which has been associated with drug resistance. Combination of an AXL inhibitor or AXL knockdown with a ROS1 inhibitor partially reversed resistance.

**Conclusions:**

In summary, we demonstrate that AXL overexpression is a mechanism of intrinsic resistance to ROS1 inhibitors.

## Graphical abstract

The left panel shows AXL mediates resistance to ROS1 inhibitor monotherapy through AKT signaling. The right panel shows a combination therapy of ROS1 and AXL inhibitors blocks AKT signaling, thus leading to tumor cell death.

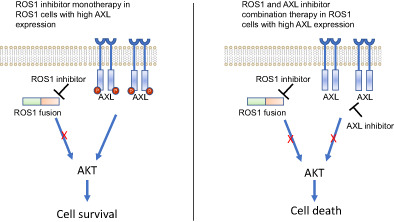

## INTRODUCTION

Treatment of non‐small cell lung cancer (NSCLC) has evolved rapidly in the era of precision medicine, with approximately 70%–75% of patients now having identifiable oncogene drivers.^1^ Many of these drivers, such as activated epidermal growth‐factor receptor (EGFR) or gene rearrangements involving anaplastic lymphoma kinase (ALK) or ROS proto‐oncogene 1 (ROS1) have targeted therapies approved for treatment. Treatment with EGFR, ALK and ROS1 targeted therapy has dramatically prolonged patient survival and quality of life compared to chemotherapy, but resistance to targeted therapy still poses a major clinical challenge.^2^, ^3^

Within lung adenocarcinoma, *ROS1* gene fusions make up approximately 1%–2% of NSCLC cases.^1^ The majority of patients harboring *ROS1* gene fusions achieve a response to the ROS1 tyrosine kinase inhibitors (TKI) crizotinib and entrectinib, with objective response rates of 72% and 77%, respectively.^3^, ^4^ While intrinsic, or de novo, resistance to targeted therapy in lung cancer is rare and not well understood, acquired resistance to TKIs is similar for all oncogene driven NSCLC and can arise by mutations to the kinase domain of the target, activation of bypass signaling pathways or cellular state transformation, such as epithelial to mesenchymal transition (EMT). Acquired resistance due to bypass signaling or cellular transformation can be achieved by many different proteins or cellular programs including EGFR and KIT, but AXL tyrosine kinase is a mediator of resistance in multiple cancers, including NSCLC.^5^, ^6^, ^7^, ^8^ AXL can activate bypass signaling, plays a role in immune suppression, and is part of an EMT gene signature associated with resistance.^5^, ^7^, ^8^, ^9^, ^10^, ^11^, ^12^ Here, we report a unique case of a patient with a *ROS1* gene fusion and intrinsic resistance to ROS1 targeted therapy driven by expression of AXL prior to starting ROS1 targeted therapy.

## METHODS

### Clinical testing

Patient samples were tested by the CLIA certified Colorado Genetics Laboratory (CGL) and Colorado Molecular Correlates Laboratory (CMOCO). Tumor testing was performed using a customized version of the ArcherDx VariantPlex Solid Tumor and FusionPlex library preparation kits (ArcherDx). Libraries were sequenced on the Illumina platform, and the data was processed using the ArcherDx Analysis packages. For cfDNA analysis, we used the Guardant360 NGS assay (Illumina) using hg19 as the reference genome.

### Cell line derivation and reagents

CUTO33 cell line derivation was performed as previously described following Institutional Review Board approved informed consent of the patients.^13^ All cell lines were cultured in RPMI1640 (ThermoFisher) with 10% FBS (ThermoFisher) at 37° with 5% CO_2_. All drugs were purchased from Selleck Chemicals.

### Proliferation assays

Cells were seeded at the appropriate density (determined empirically) in 96 well plates and treated with the indicated concentration of drug the following day. After 72 h MTS (CUTO28, CUTO33, HCC78 and HCC78‐TR) or CyQUANT reagent (CUTO23, CUTO27, and CUTO37) were added as per the manufacturers’ recommendations (Promega/ThermoFisher Scientific) and absorbance was measured on a microplate reader (BioTek). IC_50_ values were calculated using nonlinear regression curve fitting in GraphPad Prism. Combination index was calculated based on the IC_50_ using the Chou‐Talay method.^14^

### Western blot

Western blot was performed as previously described.^13^ All drug treatments were done at the indicated doses for 2 h. Antibodies used were ROS1 (D4D6®), p‐ROS1 Y2274 (3078), AXL (8661), MET (3148S), p‐MET Y1234/1235 (3077S), p‐SHP2 Y542 (3751S), ERK1/2 (4696), p‐ERK1/2 T202/Y204 (4370), Akt (2920), p‐Akt S473 (4060) from Cell Signaling Technology; SHP2 (610622) from BD Biosciences; and GAPDH (MAB374) from Millipore.

### AXL knockdown

We transfected 50 nM AXL siRNA pool (Horizon) into the CUTO33 cells using Lipofectamine transfection reagent, as per the manufacturer's protocol. The following day cells were plated for MTS assay and protein analysis, which was done at 48 h after transfection.

### Colony formation assay

A total of 300 cells/mL were seeded in six‐well plates which were treated the following day with the indicated drugs. Drug media was refreshed every 3 days for the duration of the 14‐day assay. All treatments were done in triplicate and the experiment was repeated for three biological replicates.

### RNA sequencing

RNA was isolated from triplicate biological samples using the RNEasy Kit (Qiagen) and submitted for RNA sequencing and annotation by Novogene.^15^

### Single‐sample GSEA for EMT gene signature assessment

Since single‐sample GSEA analysis calculates an enrichment score for each gene set by comparing gene expression within an individual sample, FPKM values from the RNA‐seq data with gene length normalized were used for this type of analysis. Lowly expressed genes with the sum of FPKM counts across cell lines below 1 were excluded. Additional duplicated genes were filtered out by only retaining the ones with a higher sum value of FPKM counts across cell lines. ssGSEA analyses were conducted by R package GSVA version 1.46.0 and EMT‐related gene sets were obtained from MSigDB using R package msigdbr version 7.5.1.^16^ ssGSEA scores for each gene set were scaled across different ROS1 lines and presented in a heatmap using R package pheatmap version 1.0.12.

## RESULTS

### Patient case presentation

**FIGURE 1:**
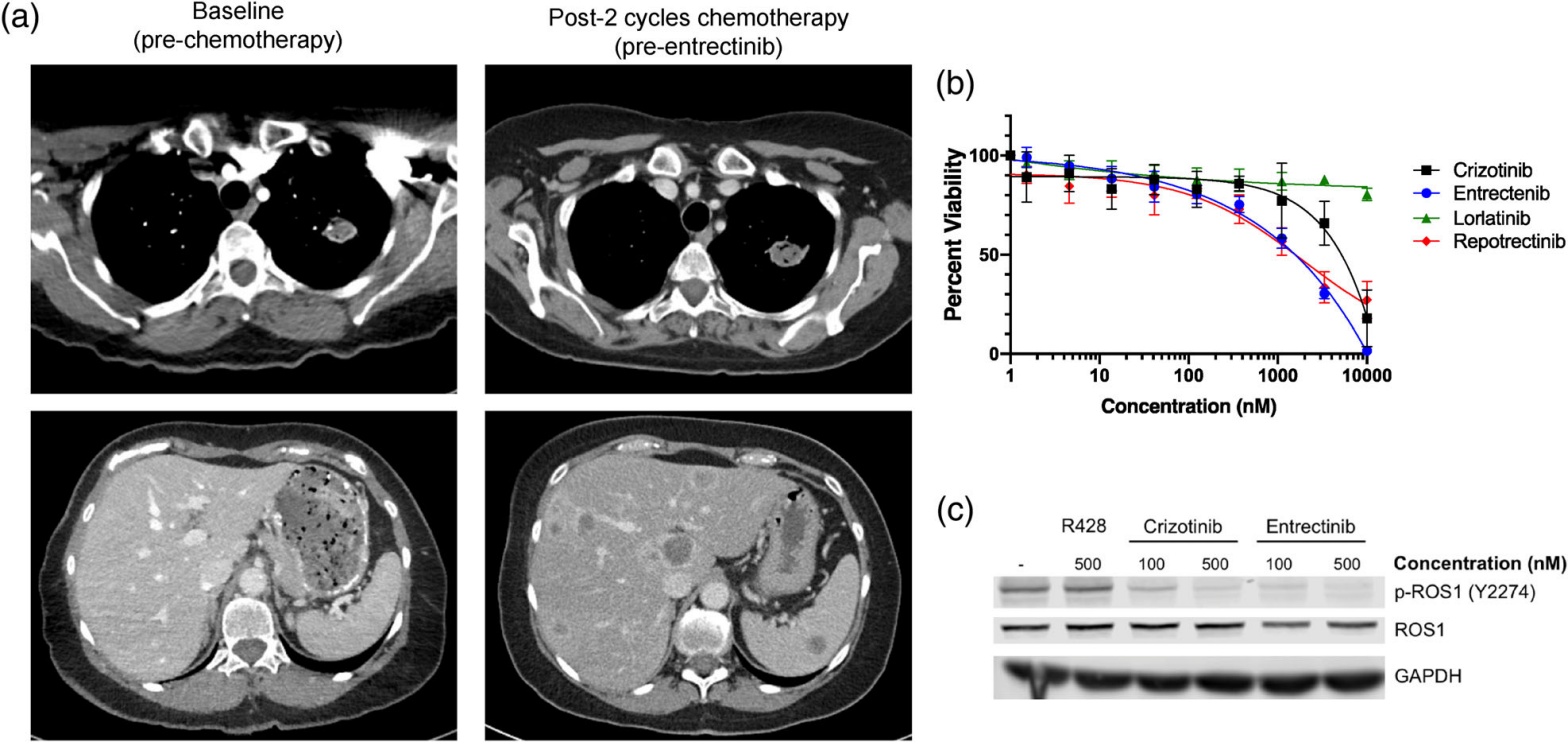
Derivation of a ROS1 inhibitor resistant cell line from a patient with intrinsic resistance. (a) Computed tomography (CT) scan of the patient prior to and after two cycles of chemotherapy. (b) Cell viability analysis with ROS1 inhibitors. CUTO33 cells were treated with the indicated concentration of drugs for 72 h and proliferation was measured by MTS assay. Showing the mean+/− SD, *n* = 3 biological replicates. (c) Inhibition of ROS1 phosphorylation. CUTO33 cells were treated with DMSO or the indicated concentrations of ROS1 inhibitors for 2 h prior to cell lysis and protein analysis by western blot. Representative images, *n* = 2.

The patient was a never smoking 65 year‐old female who presented with unilateral left vision loss and was found to have two ring enhancing lesions in the bilateral parietal lobes with further work‐up identifying a left lung mass. Percutaneous biopsy of the lung mass demonstrated poorly differentiated adenocarcinoma with TTF‐1 expression consistent with lung origin, but insufficient tissue for molecular testing. The patient received stereotactic radiosurgery to the brain lesions and initiated treatment with carboplatin and pemetrexed. Following two cycles of chemotherapy, the patient sought a second opinion at the University of Colorado, where imaging confirmed the presence of brain metastasis and showed new lymphadenopathy, splenic metastases and diffuse metastases in the liver (Figure 1a). Analysis of a ctDNA blood sample was performed using the Guardant360 assay and revealed a *CD74‐ROS1* gene fusion, a *TP53* mutation (W146*), and multiple variants of unknown significance. Tumor testing on a biopsy from a liver metastasis confirmed the *CD74‐ROS1* (*CD74* exon 6 to *ROS1* exon 34) fusion as well as the *TP53* mutation. The patient was enrolled on a phase II clinical trial of entrectinib (NCT02568267), but unfortunately died before follow‐up imaging due to rapid disease progression.

### Initial characterization of the CUTO33 cell line

**FIGURE 2:**
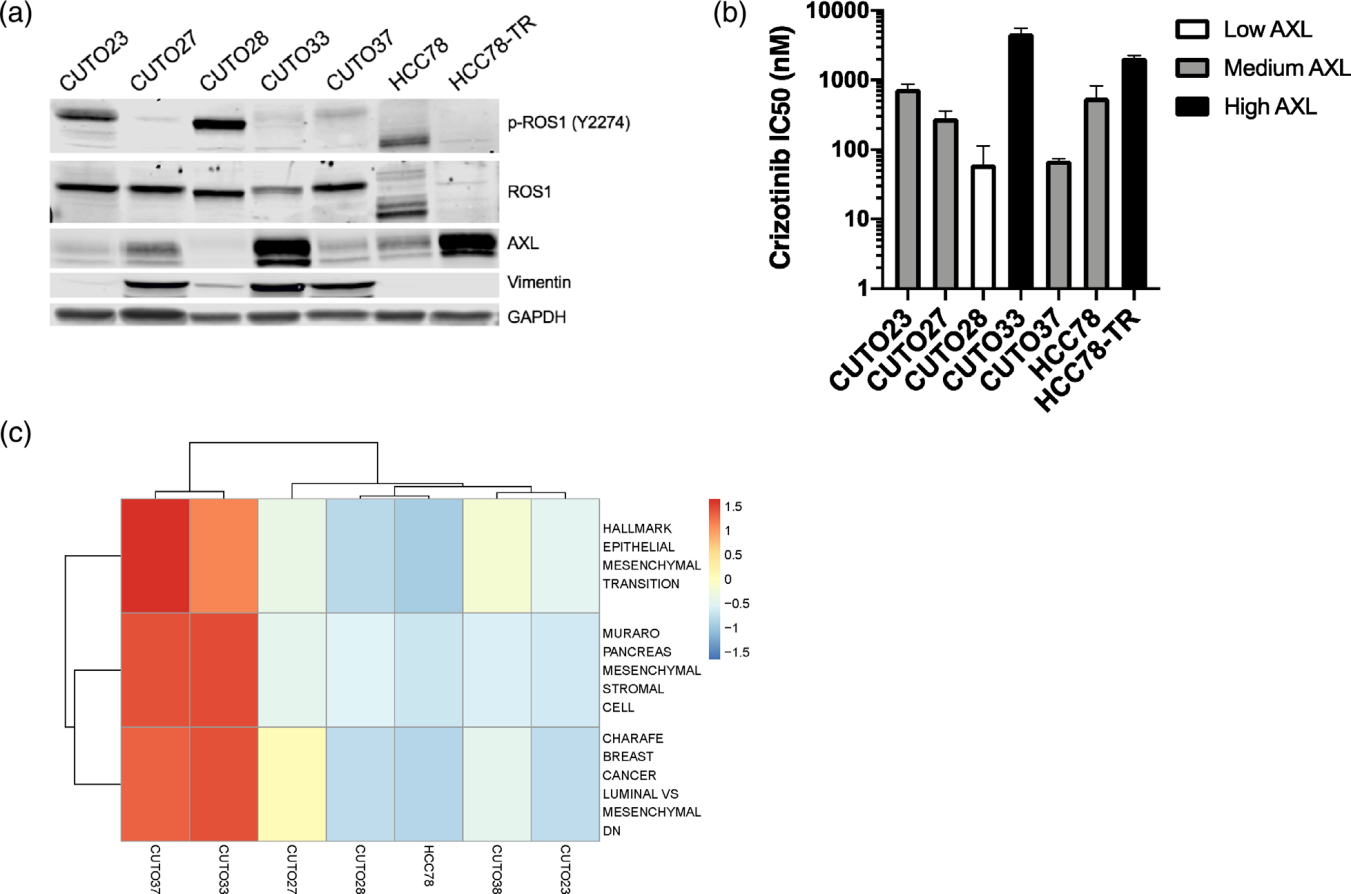
CUTO33 expresses high levels, which is associated with resistance to ROS1 inhibitors. (a) ROS1 and EMT protein expression in ROS1 fusion non‐small cell lung cancer (NSCLC) cell lines. Cell lysates were made from ROS1 fusion positive NSCLC cell lines and protein was analyzed by western blot for the indicated proteins. (b) Correlation of crizotinib sensitivity with AXL protein expression. Half‐maximal inhibitory concentrations (IC_50_s) of crizotinib in the panel of ROS1 fusion cell lines were measured by MTS assay and calculated using nonlinear regression in GraphPad Prism. Showing the mean+/− SD, *n* = 3 biological replicates. (c) Heatmap of single‐sample GSEA (ssGSEA) scores for EMT‐related gene sets scaled across different ROS1 cell lines. ssGSEA analyses were performed to calculate enrichment scores for EMT‐related gene sets for each individual cell line RNA‐seq data. Red color in the heatmap indicates an enrichment in mesenchymal gene signature expression.

The patient‐derived cell line generated from the liver metastasis biopsy, CUTO33, maintained the *CD74‐ROS1* fusion as well as resistance to multiple ROS1 TKIs including crizotinib, entrectinib, repotrectinib and lorlatinib (Figure S1a,b). Protein analysis revealed that ROS1 activity was inhibited by the ROS1 TKIs crizotinib and entrectinib, consistent with the lack of a kinase domain mutation in ROS1 (Figure 1c).^17^ Because primary resistance to ROS1 TKI is a rare occurrence, we compared the CUTO33 cell line to a panel of patient‐derived ROS1 fusion cell lines (Figure 2a). All the cell lines have a *CD74‐ROS1* fusion except CUTO28, which harbors a *TPM3‐ROS1* fusion, and HCC78 and HCC78‐TR which have *SLC34A2‐ROS1*.^13^ There was a range of sensitivity to crizotinib, with CUTO33 and HCC78‐TR (TAE684 resistant cell line derived from the HCC78) being resistant (Figure 2b).^13^ The HCC78‐TR cell line was previously reported to lose expression of the ROS1 fusion protein, while the other cell lines showed similar expression of the fusion, but varied in their levels of ROS1 phosphorylation.^13^ As AXL has been reported to drive acquired resistance to TKIs in NSCLC we evaluated its protein expression, as well as expression of the EMT marker vimentin to determine if an EMT phenotype could explain the resistance observed in the CUTO33 cell line.^5^, ^10^, ^18^ We found a range of AXL expression in the ROS1 fusion cell lines, but the CUTO33 and HCC78‐TR cell lines had the highest expression of AXL and the lowest basal levels of ROS1 phosphorylation (Figure 2a). Variable vimentin expression was observed in both sensitive and resistant ROS1 cell lines and did not correlate with sensitivity to ROS1 inhibition.

To broaden the evaluation of EMT signature gene expression and ask whether EMT correlates with ROS1 inhibitor sensitivity, we conducted RNA‐seq for a panel of ROS1 cell lines and employed single‐sample GSEA analysis to calculate enrichment scores for EMT‐related gene sets for each cell line.^19^ Despite a well‐described association between AXL and EMT,^5^, ^8^, ^10^, ^11^ EMT signatures might not be a good indicator of ROS1 inhibitor resistance since the gene expression of CUTO37, a crizotinib‐sensitive cell line was also enriched in a high mesenchymal program (Figure 2c). Overall, those data suggest AXL expression, but not EMT signature, may be associated with cellular responses to ROS1 inhibition.

### Resistance to crizotinib is driven by AXL in the CUTO33 cell line

**FIGURE 3:**
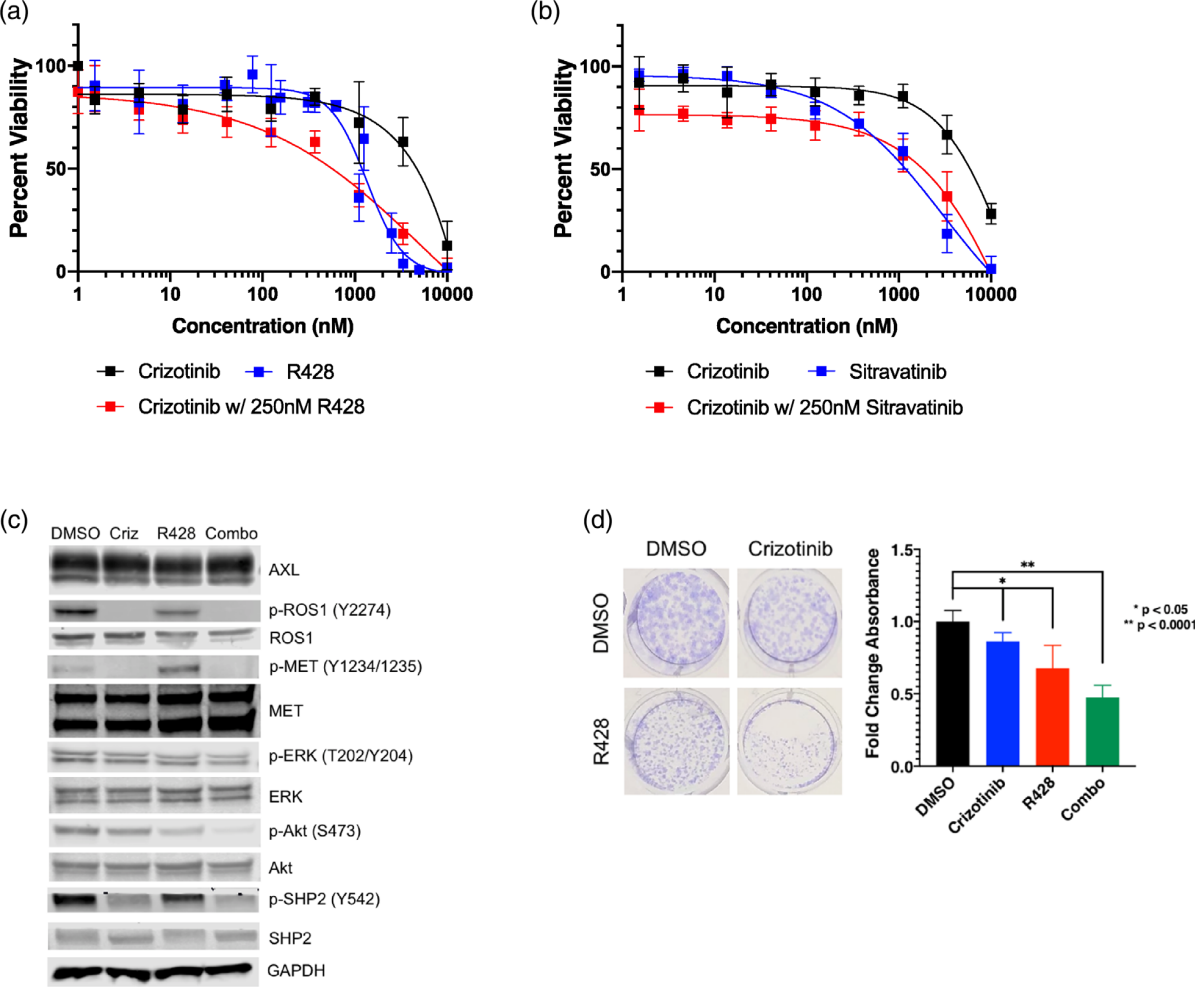
The CUTO33 cell line is resistant to single agent ROS1 inhibition, but sensitive to AXL combination therapy. (a,b) AXL inhibition with R428 (a) or sitravantinib (b) sensitizes CUTO33 cells to crizotinib. CUTO33 cell viability following treatment with the indicated concentration of drugs for 72 h and proliferation was measured by MTS assay. Showing the mean+/− SD, *n* = 3 biological replicates. (c) Downstream signaling changes following ROS1 and AXL inhibition. CUTO33 cells were treated with DMSO or the indicated concentrations of ROS1 inhibitors for 2 h prior to cell lysis and protein analysis by western blot. (d) R428 and crizotinib treatment inhibits CUTO33 long‐term cell proliferation. CUTO33 cells were plated at a low density and treated with either 500 nM crizotinib, 500 nM R428 or the combination every 3 days for a total of 14 days and then stained with crystal violet and measured using a Synergy2 Biotek plate reader. Showing the mean+/− SD, *n* = 3 biological replicates.

AXL has previously been described to have broad tumor promoting functions, therefore several inhibitors of AXL have been developed for the clinic, such as R428 and sitravatinib, which we used to evaluate the role of AXL in driving resistance to ROS1 inhibition in CUTO33.^20^, ^21^ Combining crizotinib with either AXL inhibitor was strongly synergistic, reducing the IC_50_ from 6180 to 467 nM when combined with R428, and 750 nM when combined with sitravatinib, with combination indices of 0.394 and 0.608 respectively (Figure 3a,b). Treatment with R428 effectively inhibited AXL phosphorylation as low as 100 nM (Figure S1b). When assessing downstream signaling changes, we found that crizotinib alone could not block activation of the extracellular signal regulated kinase (ERK) or protein kinase B (Akt), despite strong inhibition of phosphorylation of ROS1 (Figure 3c). Crizotinib treatment also inhibited hepatocyte growth factor receptor (MET) activation, suggesting intrinsic resistance of the CUTO33 cell line to ROS1 inhibitors was not driven by MET (Figure 3c). R428 treatment alone partially inhibited Akt activation, which was almost fully inhibited when combined with crizotinib treatment along with a moderate reduction in ERK activation (Figure 3c). We also saw a reduction in Akt phosphorylation upon combination of crizotinib with AXL knockdown by siRNA (Figure S1c). Phosphorylation of the protein tyrosine phosphatase nonreceptor 11 (SHP2), which activates the mitogen activated protein kinase pathway (MAPK) downstream of receptor tyrosine kinases such as ROS1, has been reported to drive resistance via reactivation of the MAPK pathway.^22^ We found SHP2 activation was sensitive to crizotinib treatment but not inhibition of AXL with R428 (Figure 3c). The CUTO33 cell line showed no sensitivity to either SHP099 or RMC‐4550, two specific inhibitors of SHP2, indicating that while ROS1 is still actively signaling in the CUTO33 cells, oncogenic rewiring has maintained MAPK pathway activation independent of ROS1 and SHP2 (Figure S1d).^22^, ^23^ Finally, a combination of crizotinib with R428 was able to significantly reduce colony formation (Figure 3d). Together with the synergistic inhibition of cell viability when we combined crizotinib with R428, this suggests that the intrinsic resistance to ROS1 inhibitors in CUTO33 cell was driven by AXL‐mediated bypass signaling primarily via the Akt pathway.

## DISCUSSION

Treatment of acquired resistance in NSCLC has benefited from the development of next generation inhibitors that overcome resistance mutations or identification of resistance mediators, like MET, which can in turn be treated with targeted therapies or combination therapies. For NSCLC patients with ROS1+ (or other oncogene subtypes) NSCLC whose tumors display intrinsic resistance to targeted therapy however, there are few treatment options beyond standard chemotherapy. Due to the rare nature and lack of appropriate models, studying and understanding mechanisms of intrinsic resistance remains a challenge. The derivation of a ROS1 cell line from a patient who later was found to demonstrate intrinsic resistance with a ROS1 inhibitor afforded us the opportunity to study mechanisms of intrinsic resistance.

AXL has been primarily studied in the context of acquired resistance, with only a few reports of elevated AXL expression driving intrinsic or early resistance to targeted therapies in glioblastoma, EGFR mutation positive NSCLC, and melanoma.^24^, ^25^, ^26^ Consistent with the idea that there are common mediators of resistance to therapies regardless of tumor type, this case highlights the potential for AXL to also drive intrinsic resistance to ROS1 inhibitors in NSCLC.^27^ The data presented here comprise the first report detailing the mechanistic underpinnings of primary resistance to ROS1 inhibition that is driven by AXL and presents a new therapeutic strategy for patients resistant to ROS1 targeted therapy.

The patient‐derived cell line, CUTO33, exhibited primary resistance to ROS1 inhibitors, and similar to a prior report in a ROS1 model, SHP2 activation did not mediate bypass RTK signaling.^28^ Combination of crizotinib and the AXL specific inhibitor R428 synergized to reduce cell viability and proliferation in the CUTO33 cell line. The CUTO33 and HCC78‐TR cell lines also shared low levels of ROS1 phosphorylation, suggesting resistance to ROS1 inhibitors driven by bypass signaling is associated with decreased activation of ROS1, although the mechanism underpinning this remains poorly understood. A follow up study by our lab on the HCC78‐TR cell line revealed that AXL and EGFR were driving resistance in this cell line, and combination of AXL and EGFR TKIs inhibited proliferation, providing further support for targeting AXL in select ROS1 TKI resistant patients.^29^ Although AXL has been reported to be associated with EMT, we did not observe a strong correlation between EMT gene signature expression and cellular resistance to ROS1 inhibition. Instead, all the cell lines with primary or acquired resistance to ROS1 TKI have high levels of AXL expression, suggesting that AXL expression could be a predictive biomarker for resistance to ROS1 inhibitors. This study supports AXL as a mediator of intrinsic resistance to ROS1 targeted therapy and highlights the need for further studies on AXL‐targeted therapies in ROS1 TKI resistant NSCLC.

## AUTHOR CONTRIBUTIONS

**Tara L. Peters:** Conceptualization, Formal analysis, Funding Acquisition, Investigation, Methodology, Visualization, Writing – Original draft preparation, Writing – review & editing; **Nan Chen:** Data curation, Formal analysis, Software, Visualization, Writing – review & editing; **Logan C. Tyler:** Data curation, Investigation; **Anh T. Le:** Resources; **Anastasios Dimou:** Conceptualization, Investigation; **Robert C. Doebele:** Conceptualization, Funding Acquisition, Project administration, Resources, Writing – review & editing.

## Supporting information


**Figure S1.** (A) Detection of *CD74‐ROS1* fusion by next generation sequencing. Integrated genome viewer of the CUTO33 read pile up at the *CD74* exon 6 and *ROS1* exon 34 regions where the gene rearrangement occurred. (B) AXL phosphorylation is inhibited by R428 treatment. CUTO33 cells were treated with the indicated concentrations of R428 for 2 h and pulsed for 15 min with 2 mM NaOV_4_ before lysis to detect AXL phosphorylation sites. Representative images, *n* = 2 biological replicates. (C) AXL knockdown decreases Akt and ERK activation. CUTO33 cells were transfected with control (siNT) or AXL targeting siRNA for 48 h before being treated with 500 nM crizotinib for 2 h and lysed to detect protein expression. Representative images, *n* = 2 biological replicates. (D) CUTO33 are not sensitive to SHP2 inhibition. CUTO33 cells were treated with the indicated concentration of drugs for 72 h and viability was measured by MTS assay. Showing the mean+/− SD, *n* = 3 biological replicates.

## Data Availability

RNA‐seq data for a panel of 7 ROS1 cells was deposited in GEO with the accession number GSE239844.
