# Supplementary figures and images for: Intrinsic resistance to ROS1 inhibition in a patient with CD74‐ROS1 mediated by AXL overexpression
Source: Thorac Cancer. 2023 Sep 19;14(33):3259–65. doi: 10.1111/1759-7714.15116 (PMC10665781; doi:10.1111/1759-7714.15116)

## Slide 1
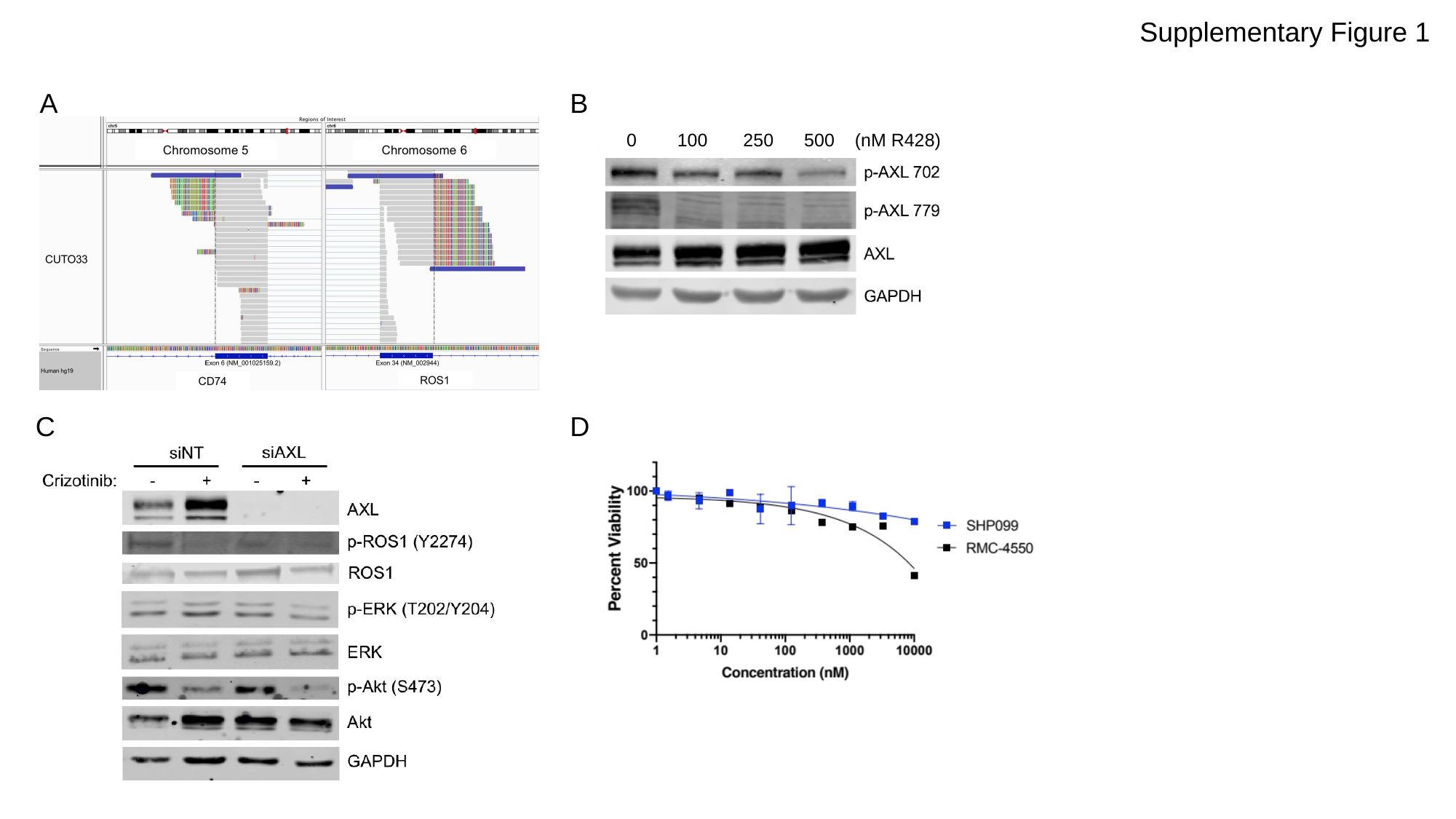

Supplementary Figure 1
A
B
 0 100 250 500 (nM R428)
C
D

Supplement: Supplementary file 1 — Figure S1. (A) Detection of CD74‐ROS1 fusion by next generation sequencing. Integrated genome viewer of the CUTO33 read pile up at the CD74 exon 6 and ROS1 exon 34 regions where the gene rearrangement occurred. (B) AXL phosphorylation is inhibited by R428 treatment. CUTO33 cells were treated with the indicated concentrations of R428 for 2 h and pulsed for 15 min with 2 mM NaOV4 before lysis to detect AXL phosphorylation sites. Representative images, n = 2 biological replicates. (C) AXL knockdown decreases Akt and ERK activation. CUTO33 cells were transfected with control (siNT) or AXL targeting siRNA for 48 h before being treated with 500 nM crizotinib for 2 h and lysed to detect protein expression. Representative images, n = 2 biological replicates. (D) CUTO33 are not sensitive to SHP2 inhibition. CUTO33 cells were treated with the indicated concentration of drugs for 72 h and viability was measured by MTS assay. Showing the mean+/− SD, n = 3 biological replicates. [file TCA-14-3259-s001.pptx]
